# Facial emotion expressivity in patients with Parkinson’s and Alzheimer’s disease

**DOI:** 10.1007/s00702-023-02699-2

**Published:** 2023-10-07

**Authors:** Antonio Cannavacciuolo, Giulia Paparella, Martina Salzillo, Donato Colella, Marco Canevelli, Davide Costa, Daniele Birreci, Luca Angelini, Andrea Guerra, Lucia Ricciardi, Giuseppe Bruno, Alfredo Berardelli, Matteo Bologna

**Affiliations:** 1 IRCCS Neuromed Pozzilli (IS), Pozzilli, Italy; 2 Department of Human Neurosciences, Sapienza University of Rome, Viale dell’Università, 30, 00185 Rome, Italy; 3 Aging Research Center, Department of Neurobiology, Care Sciences and Society, Karolinska Institutet and Stockholm University, Stockholm, Sweden; 4 Parkinson and Movement Disorder Unit, Study Center on Neurodegeneration (CESNE), Department of Neuroscience, University of Padua, Padua, Italy; 5 St George’s, University of London and St George’s University Hospitals NHS Foundation Trust, Institute of Molecular and Clinical Sciences, Neurosciences Research Centre, Cranmer Terrace, London, SW17 0QT UK

**Keywords:** Parkinson’s disease, Alzheimer’s disease, Emotion processing, Facial expressivity

## Abstract

Parkinson’s disease (PD) and Alzheimer’s disease (AD) are neurodegenerative disorders with some overlapping clinical features. Hypomimia (reduced facial expressivity) is a prominent sign of PD and it is also present in AD. However, no study has experimentally assessed hypomimia in AD and compared facial expressivity between PD and AD patients. We compared facial emotion expressivity in patients with PD, AD, and healthy controls (HCs). Twenty-four PD patients, 24 AD patients and 24 HCs were videotaped during neutral facial expressions and while posing six facial emotions (anger, surprise, disgust, fear, happiness, and sadness). Fifteen raters were asked to evaluate the videos using MDS-UPDRS-III (item 3.2) and to identify the corresponding emotion from a seven-forced-choice response format. We measured the percentage of accuracy, the reaction time (RT), and the confidence level (CL) in the perceived accuracy of the raters’ responses. We found the highest MDS-UPDRS 3.2 scores in PD, and higher in AD than HCs. When evaluating the posed expression captures, raters identified a lower percentage of correct answers in the PD and AD groups than HCs. There was no difference in raters’ response accuracy between the PD and AD. No difference was observed in RT and CL data between groups. Hypomimia in patients correlated positively with the global MDS-UPDRS-III and negatively with Mini Mental State Examination scores. PD and AD patients have a similar pattern of reduced facial emotion expressivity compared to controls. These findings hold potential pathophysiological and clinical implications.

## Introduction

Parkinson’s disease (PD) and Alzheimer’s disease (AD) are two of the most common neurodegenerative disorders with some overlapping clinical features (Martin 1999; Postuma et al. 2015; Schirinzi et al. 2020; Dubois et al. 2021). Cognitive disturbances of varying severity, including dementia, can often be present in PD (Baiano et al. 2020; Goldman and Sieg 2020; Sousa-Fraguas et al. 2022). On the other hand, clinical signs of parkinsonism, including bradykinesia, postural instability, gait abnormalities and axial rigidity have been frequently described in AD (Ellis et al. 1996; Scarmeas et al. 2004; Bologna et al. 2020). In light of the occurrence of common symptoms in AD and PD, recent studies have suggested that these two conditions might not be completely distinct pathological entities but rather part of a phenotypic continuum of degenerative processes that can partially involve the same brain areas (Perl et al. 1998; Pan et al. 2019; Schirinzi et al. 2020).

Hypomimia is a common sign of PD, and it has been defined as reduced facial expressiveness, which can affect spontaneous facial movements like blinking or other facial movements, as well as the expression of emotions (Jankovic 2008; Bologna et al. 2013; Ricciardi et al. 2017, 2020). In PD, hypomimia has been described in up to 70% of cases, and it can often be a prominent manifestation from the very early stages of the disease (Ricciardi et al. 2020; Sampedro et al. 2022). Hypomimia in PD can be correlated with the severity of other axial signs and symptoms, it can be associated with apathy (Bologna et al. 2013; Ricciardi et al. 2020), and it has also been identified as one of the main determinants of impaired quality of life in patients (Sampedro et al. 2022; Argaud et al. 2018a; Cacabelos 2017; Pegolo et al. 2022; Ma et al. 2019). Hypomimia in PD has been characterized in a series of clinical and experimental studies (Bologna et al. 2013; Argaud et al. 2018a; Sampedro et al. 2022). Several clinical studies have highlighted hypomimia in AD (Ellis et al. 1996; Tsolaki et al. 2001; Tosto et al. 2016). However, as opposed to PD, only a few experimental works have been interested in the study of hypomimia in AD, some of which have focused specifically on the facial expression of pain (Seidl et al. 2012; Burton and Kaszniak 2006; Beach et al. 2016; Lautenbacher and Kunz 2017). Importantly, to date, no studies have specifically compared facial emotion expressivity in patients with PD and AD.

From a pathophysiological standpoint, recent clinical and neuroimaging studies in PD have demonstrated the relationship of hypomimia with the central dopaminergic deficit and other specific aspects related to the dysfunction of emotion processing, such as emotional valence processing (Gerardin et al. 2003; Bologna et al. 2013; Ricciardi et al. 2020; Comon et al. 2022). Notably, a central dopaminergic dysfunction has been demonstrated by several studies also in patients with AD (Perez et al. 2005; Mitchell et al. 2011; Martorana and Koch 2014; Pan et al. 2019). Furthermore, both PD and AD are characterized by the involvement of other brain areas participating in emotion processing (Perez et al. 2005; Pan et al. 2019; Fádel et al. 2019; Bell et al. 2019; Ferrari et al. 2021). Given these pathophysiological similarities between PD and AD, it is possible that the two conditions also demonstrate a degree of similarity in altered facial expressiveness. A detailed investigation of this issue could provide useful information for a correct pathophysiological framing of this condition.

Thus, in the present study, we aimed to investigate facial emotion expressivity in patients with PD and AD compared to a control group of healthy individuals. We also investigated the possible relationship between facial emotion expressivity in PD and AD patients and the clinical features of both pathological conditions. The findings of this study hold potential pathophysiological implications. Moreover, the results could have significant clinical implications, particularly distinguishing between PD and AD during the diagnostic process.

## Materials and methods

### Participants

This study was conducted at the Department of Human Neurosciences, Sapienza University of Rome. A total of seventy-two participants were recruited: 24 patients diagnosed with PD according to the Movement Disorder Society criteria (Movement Disorder Society Task Force on Rating Scales for Parkinson’s Disease 2003), 24 patients diagnosed with AD based on the probability criteria of the National Institute on Aging-Alzheimer’s Association (McKhann et al. 2011), and 24 age- and gender-matched healthy controls (HCs). We excluded from the study participants with other neurodegenerative or secondary dementia, as well as those with atypical or secondary parkinsonism. We also excluded patients with PD who had developed dyskinesia from our study, due to the potential confounding influence of facial dyskinesia on the assessment of facial expressivity. Patients were studied in the morning before the intake of their usual therapy. In patients, information on demographic data, medical history, and disease progression was collected through direct interviews with patients and caregivers. We also conducted the following assessments: general and neurological physical examinations, administration of standardized clinical scales, including the motor section of the Movement Disorders Society-Sponsored Revision of the Unified Parkinson’s Disease Rating Scale (MDS-UPDRS-III) (Movement Disorder Society Task Force on Rating Scales for Parkinson’s Disease 2003; Goetz et al. 2008; Antonini et al. 2013), the Apathy Evaluation Scale (AES) (Marin et al. 1991), the Beck Anxiety Inventory (BAI) (Beck et al. 1988), the Frontal Assessment Battery (FAB) (Dubois et al. 2000), the Mini-Mental State Examination (MMSE) (Tombaugh and McIntyre 1992), the Beck Depression Inventory (BDI) (Beck et al. 1961), as well as the assessments of Activities of Daily Living (ADL) (Katz et al. 1963) and Instrumental Activities of Daily Living (IADL) (Lawton and Brody 1969). All subjects provided informed consent and agreed to undergo the procedures outlined in the study, which received ethical approval from the Local Ethics Committee. The study was conducted following the Declaration of Helsinki.

### Video recordings and facial expressions evaluation

A standardized video recording protocol was used for each participant during one session (Ricciardi et al. 2017). Participants were seated in a chair without head support, with feet flat on the floor and hands resting on the chair armrests. The camera captured only the upper body, including the head, hair, and shoulders. The assessment included three parts: (i) Video recording of the static facial expression at rest (60-s duration), and (ii) Video posing the six primary facial expressions (anger, surprise, disgust, fear, happiness, and sadness) (Emotion Expressivity Task). The emotions were randomly elicited by the evaluator (Ricciardi et al. 2015, 2017). Each subject was asked to describe by example a setting of each emotion through images from daily life (“Tell me about an episode in which you experienced happiness, fear, anger, etc.”) to ensure proper understanding and execution of the task at hand. The most expressive pictures for each emotion recording were selected from the videos. In detail, according to a protocol previously used in another study, we selected a short 4-s sequence, then divided it into four sections of equal duration, and from these, we extracted the image that best expressed the required emotion for a total of 504 pictures (Ricciardi et al. 2017).

Fifteen neurologists (6 females and 9 males) with expertise in movement disorders and/or dementia were asked to evaluate the video recordings and captures. We employed a validated test to control for a possible deficit in facial emotion recognition in the raters. i.e., the ‘Ekman 60-Faces Test’ (Emotion Recognition Task). The test consisted of 60 images depicting 10 actors (6 females and 4 men) expressing six emotions (anger, surprise, disgust, fear, happiness and sadness) (Anon 1976). Each rater viewed the images on a computer screen and selected the corresponding emotion from six alternatives. They received one point for each correct answer and zero points for incorrect answers, with a maximum score of 60 points indicating the best possible performance. Cut-off values were established using normative data (Dodich et al. 2014). All raters scored above the cut-off (mean and standard deviation of the scores 87.34 ± 4.74%). Afterward, raters evaluated the video recordings and the pictures of the 72 participants. During the evaluation, raters were seated in front of a high-resolution PC screen. They first evaluated the 72 videos of the participants’ facial expressions, presented in their entire duration (60 s). Raters were asked to score the video using the MDS-UPDRS Part III, item 3.2, thus scoring facial expression from score 0 ("normal facial mimic") to score 4 (“Fixed facial expressions with lips open most of the time when the mouth is still”) (Goetz et al. 2008; Antonini et al. 2013; Ricciardi et al. 2017). We did not impose any time limit for this task, and raters could watch the videos as many times as they wished. After that, raters assessed the 504 pictures extracted from the video (Emotion Recognition Task) and identified the corresponding emotion in each picture choosing among seven options (anger, surprise, disgust, fear, happiness, sadness, or neutral). The pictures were presented to the raters if they required responding. However, raters were asked to respond as quickly as possible. Raters’ response reaction time (RT) was recorded. Each correct answer was given one point, while incorrect answers received no points. Raters were also asked to indicate their confidence level (CL) in the chosen answer on an analog scale displayed on the screen, ranging from 0 (indicating low confidence) to 10 (indicating high confidence). Notably, raters were blinded for participants’ diagnoses, and both videos and captures were presented to them in random order.

### Statistical analysis

Gender and age differences between PD and AD patients and HCs were assessed using a chi-square test and non-parametric one-way analysis of variance (ANOVA), i.e., a Kruskal–Wallis ANOVA, respectively. A Kruskal–Wallis ANOVA and Mann–Whitney *U* tests were employed to investigate potential differences in clinical scale scores between participants (Table 1). We considered each rater’s average score at the Emotion Recognition Task, and we also performed a one-way ANOVA to assess possible differences in recognition of the different emotions, using the within-subjects factor “EMOTION” (with seven levels: anger, disgust, fear, happiness, sadness, surprise, or and neutral expressions).

**Table 1:** Clinical-demographic characteristics of patients with Parkinson’s disease (PD), Alzheimer’ disease (AD) and healthy controls (HCs)

	PD (*n* = 24)	AD (*n* = 24)	HCs (*n* = 24)	*p* value
Gender	7 F	15 F	11 F	0.23
Age (years)	75.21 ± 3.4	76.5 ± 6.52	74.04 ± 6.68	0.08
MDS-UPDRS III global score	40.08 ± 17.9	6.79 ± 6.1	2.33 ± 3.2	< **0.001**
MMSE	26.08 ± 1.9	17.04 ± 6.9	27.62 ± 1.7	**0.02**
AES	42.58 ± 7.5	47.71 ± 10.1	38.79 ± 7.8	< **0.01**
BDI-II	9.42 ± 5.7	13.75 ± 7	7.17 ± 4.6	< **0.001**
BAI	10.08 ± 8	7.25 ± 8	6.12 ± 4.6	0.12
FAB	13.71 ± 3.4	9.33 ± 4.6	15.63 ± 3.8	< **0.001**
Disease duration (years)	9.54 ± 6.2	4.23 ± 3.1	–	< **0.001**

*PD* Parkinson’s Disease, *AD* Alzheimer disease, *HCs* healthy controls, *MDS-UPDRS-III* Movement Disorder Society Unified Parkinson Disease Rating Scale—Part III, *MMSE* Mini Mental State Examination, *AES* Apathy Evaluation Scale, *BDI-II* Beck Depression Inventory, *BAI* Beck Anxiety Inventory, *FAB* Frontal Assessment Battery. Significant *p* values at post-hoc comparisons are in bold

Fleiss’ K was employed to calculate the interrater agreement during the MDS-UPDRS-III item 3.2 evaluation (Facial Expression). A non-parametric one-way ANOVA was then applied to compare scores among the three groups (median values of the raters’ scores). Post hoc analyses were performed with the Mann–Whitney *U* test. Also, according to the MDS-UPDRS-III item 3.2 scores, we divided the whole sample of participants into two subgroups: ‘with hypomimia’ (MDS-UPDRS-III item 3.2 score ≥ 2), and ‘without hypomimia’ (MDS-UPDRS-III item 3.2 score < 2). We then performed between-group comparisons using unpaired *t*-tests.

To analyze the accuracy of responses during the Emotion Expressivity Task, a factorial ANOVA was performed using the between-subjects factor “GROUP” (3 levels: PD, AD, and HCs) and the within-subjects factor “EMOTION” (7 levels: anger, disgust, fear, happiness, sadness, surprise, and neutral expression). The same statistical analysis was applied to measure possible changes in the raters’ RT and CL. Post hoc analyses were performed using Bonferroni Test.

Spearman’s correlation test assessed possible relationships between demographic/clinical data and the median scores obtained at the MDS-UPDRS –III item 3.2 and during the Emotion Recognition Task (percentage of correct answers considering all raters data, i.e., overall recognition rate). Mean values ± 1 standard error of the mean (SEM) were used to present the results unless otherwise specified. Statistical significance was set at *p* < 0.05.

## Results

### Demographic and clinical data

There were no significant age differences among the three groups of participants (all *p*s > 0.05). We did not find any difference in gender distribution between PD and HCs (*p* = 0.23) and between AD and HCs (*p* = 0.24). However, a significant difference in gender distribution was observed between PD and AD (*p* = 0.02). Kruskal–Wallis ANOVA indicated a significant difference in the MDS-UPDRS-III global scores between the three groups of participants [H(2) = 55.55, *p* < 0.01]. Post-hoc comparisons showed, as expected, that PD patients had higher scores than the other two groups (both *p*s < 0.01). Conversely, AD patients had lower scores on the MMSE and FAB than PD and HCs (both *p*s < 0.01). We observed a difference in AES scores among the three groups [H(2) = 10.56, *p* < 0.01]. Post-hoc analysis revealed that AD patients had significantly higher AES scores compared to HCs (*p*s < 0.01), with no significant differences between AD and PD, nor between PD and HCs (both *p*s > 0.05). For additional information, refer to Table 1.

### Facial expression results

**Fig. 1:**
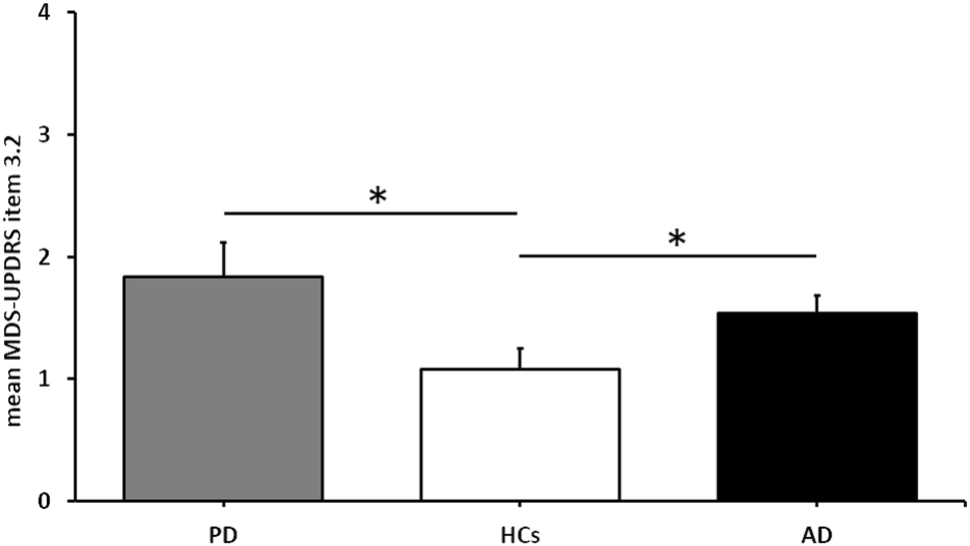
Facial expression results. Movement Disorders Society-Sponsored Revision of the Unified Parkinson’s Disease Rating Scale (MDS-UPDRS) part III, item 3.2 scores in the three group of participants as blinded evaluated by 15 raters. *PD* Parkinson’s Disease, *AD* Alzheimer disease, *HCs* healthy controls, *MDS-UPDRS-III*  Movement Disorder Society Unified Parkinson Disease Rating Scale—Part III. Asterisks indicate significant *p* values at post-hoc comparisons

The interrater’ agreement was slight (Fleiss’ K = 0.03) (Landis and Koch 1977). Kruskal–Wallis ANOVA indicated a significant difference in the MDS-UPDRS-III item 3.2 scores between the three groups of participants [H (2) = 14.69, *p* < 0.01]. Post-hoc comparisons showed higher scores in PD than HCs (*p* < 0.01), as well as higher scores in AD as compared to HCs (*p* < 0.02), with no difference between PD and AD (*p* = 0.18) (Fig. 1). By performing the subgroup analysis according to the median MDS-UPDRS-III item 3.2 score (Table 2), we found that 39 out of 72 subjects (54.2%) were included in the subgroup ‘with hypomimia’. Out of these, 19 (48.7%) were PD patients, 14 (35.9%) were AD patients, and the remaining (6 subjects, 15.4%) were HCs. In the ‘without hypomimia’ subgroup (33 subjects in total, 45.8%), most of the subjects were HCs (18 subjects, 54.5%), while 10 individuals were AD patients (30.3%), and only 5 were PD patients (15.2%). We found no difference between the two subgroups in terms of age (*p* = 0.16) and gender distribution (*p* = 0.07 by Chi-square). MMSE scores, as well as other clinical scales the scores, were also similar in the two subgroups (all *p*s > 0.05) (Table 2). Conversely, as expected the MDS-UPDRS global scores were higher in the subgroup ‘with hypomimia’ than in the subgroup ‘without hypomimia’ (p < 0.01).

**Table 2:** Clinical-demographic characteristics of the participants divided into the ‘with hypomimia’ and ‘without hypomimia’ subgroups

	With hypomimia (*n* = 39)	Without hypomimia (*n* = 33)	*p* value
Gender	14 F	19 F	0.07
Age (years)	76.13 ± 4.71	74.21 ± 6.69	0.16
MDS-UPDRS III global score	24.8 ± 22.06	9.55 ± 13.37	< **0.01**
MMSE	22.45 ± 6.19	24.15 ± 6.84	0.3
AES	44.56 ± 10.05	41.34 ± 7.75	0.13
BDI-II	12.07 ± 5.7	9.79 ± 5.45	0.08
BAI	8.87 ± 7.92	7.85 ± 4.52	0.5
FAB	12.77 ± 3.9	14.03 ± 4.36	0.2

*MDS-UPDRS-III*  Movement Disorder Society Unified Parkinson Disease Rating Scale—Part III, *MMSE*  Mini Mental State Examination, *AES*  Apathy Evaluation Scale, *BDI-II*  Beck Depression Inventory, *BAI*  Beck Anxiety Inventory, *FAB* Frontal Assessment Battery. Significant p values at post-hoc comparisons are in bold

## Emotion expressivity task

### Percentage of accuracy

Raters achieved an average score > 80% (range 72–93%) on the Ekman 60-faces test (Emotion Recognition Task). This finding demonstrates that none of the raters had a deficit in facial emotion recognition. Significant differences were found among the recognition of the different facial expressions [F (6, 84) = 6.44, *p* < 0.01]. Post-hoc analysis showed a lower percentage of correct answers in recognition of “disgust” as compared to “surprise”, “neutral expression”, and “happiness” (all *p*s < 0.01), and in recognition of “sadness” as compared to “happiness” (*p* = 0.01), “surprise” (*p* = 0.02), and “neutral expression” (*p* < 0.01).

**Fig. 2:**
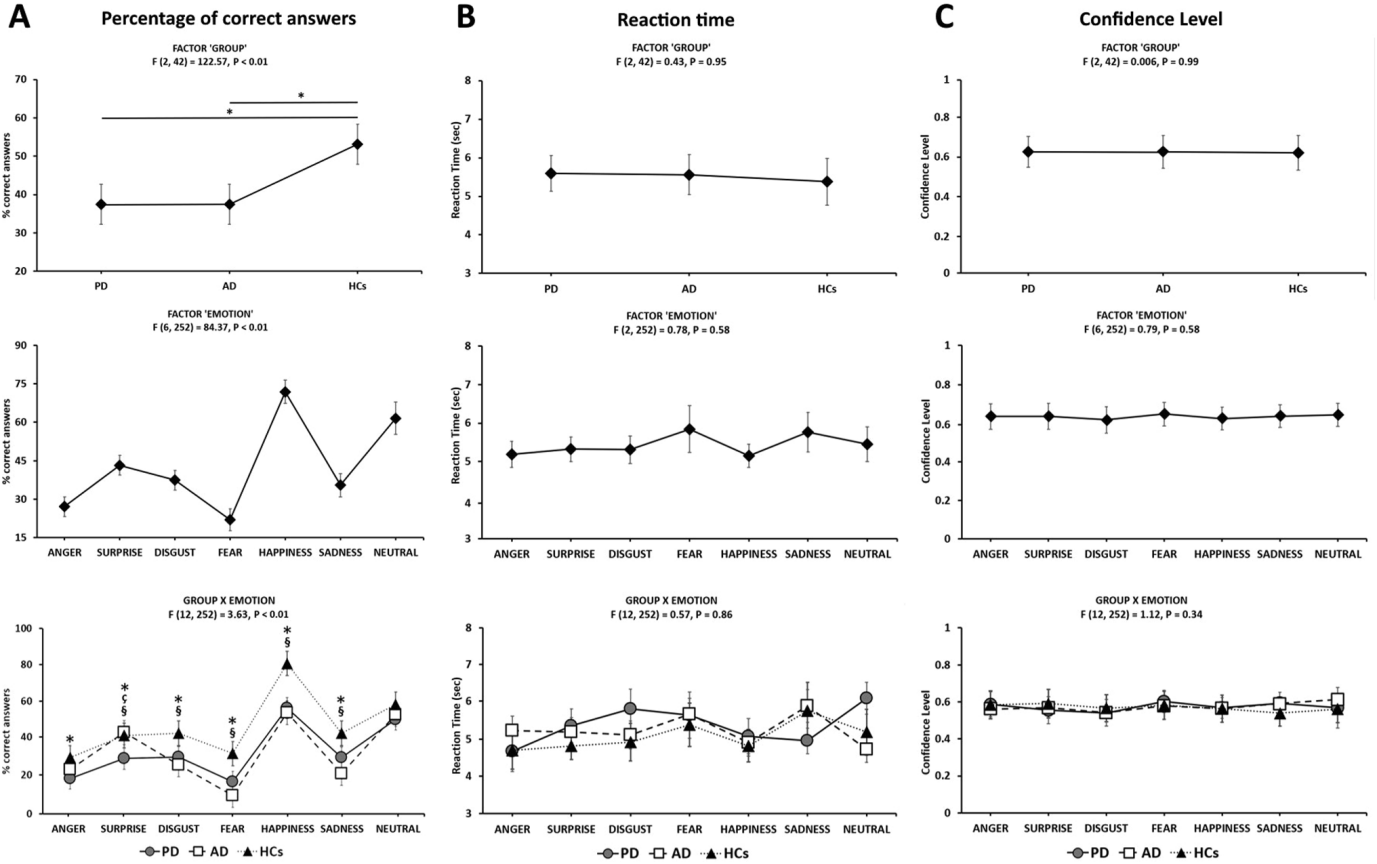
Emotion Expressivity Task. **A** Percentage of correct answers: Percentage of correct answers given by the 15 raters during the blinded evaluation of the 504 pictures extracted from the recorded video (Emotion Recognition Task). Asterisks (*) indicate significant *p* values at post-hoc comparisons between PD and HCs. Section sign (§) indicate significant p values at post-hoc comparisons between AD and HCs. C-cedilla (ç) indicate significant *p* values at post-hoc comparisons between PD and AD. **B** Reaction time: Reaction time measured during the blinded evaluation of the 504 pictures extracted from the recorded video (Emotion Recognition Task). Data from 15 raters are shown. **C** Confidence level: Confidence level in the perceived accuracy of the raters’ responses during the blinded evaluation of the 504 pictures extracted from the recorded video (Emotion Recognition Task). Data from 15 raters are shown. *PD* Parkinson’s Disease, *AD* Alzheimer disease, *HCs* healthy controls, *MDS-UPDRS-III*  Movement Disorder Society Unified Parkinson Disease Rating Scale—Part III. Asterisks indicate significant *p* values at post-hoc comparisons

The ANOVA on the Emotion Expressivity Task data revealed a significant effect of the factor “GROUP” [F (2, 42) = 22.57, *p* < 0.01]. Post-hoc analysis showed a lower percentage of correct answers in the evaluation of both AD and PD compared to HCs (both *p*s < 0.01), with no difference between AD and PD (*p* = 1). The ANOVA also revealed a significant effect of the factor “EMOTION” [F (6, 252) = 84.37, *p* < 0.01]. Post-hoc analysis showed a higher percentage of correct responses in the recognition of “happiness” and “neutral expression” as compared to all other emotions (all *p*s < 0.01), and in the recognition of “fear” as compared to all the other emotions (all *p*s < 0.01) except for “anger” (*p* = 1). Again, we found a lower percentage of correct answers in recognition of the emotion “anger” as compared to “surprise” and “disgust” (both *p*s < 0.01), and of the emotion “surprise” and “disgust” as compared to “fear” (*p* < 0.01). Finally, the ANOVA revealed a significant interaction “GROUP x EMOTION” [F (12, 252) = 3.63, *p* < 0.01]. Post-hoc analysis showed a lower percentage of correct answers in recognition of the “disgust”, “fear”, “happiness” and “sadness” emotions in PD and AD as compared to corresponding emotions in HCs (all *p*s < 0.01), in recognition of “surprise” in PD as compared to corresponding emotion in AD (*p* < 0.01) and of “anger” in PD, as compared to corresponding emotion in HCs (*p* < 0.01); (Fig. 2A).

The overall recognition rate of emotional expressions was lower for the subgroup of subjects ‘with hypomimia’ as compared to the subgroup ‘without hypomimia’ (mean ± SD: 34.41 ± 14.43 vs 47.8 ± 16.14, *p* = 0.01).

### Reaction time and confidence level

The ANOVA on RT data revealed a no significant effect for the factors "GROUP" (F (2, 42) = 0.43, *P* = 0.95), “EMOTION” [F (2, 252) = 0.78, *P* = 0.58], nor a significant interaction “GROUP × EMOTION” [F (12, 252) = 0.57, *P* = 0.86], (Fig. 2B). Similarly, the analysis on CL data did not show any significant effect of factors nor any interaction between factors: “GROUP” [F (2, 42) = 0.006, *P* = 0.99], “EMOTIONS” [F (6, 252) = 0.79, P = 0.58], “GROUP × EMOTIONS”: [F (12, 252) = 1.12, *P* = 0.34], (Fig. 2C).

### Correlation analysis

**Fig. 3:**
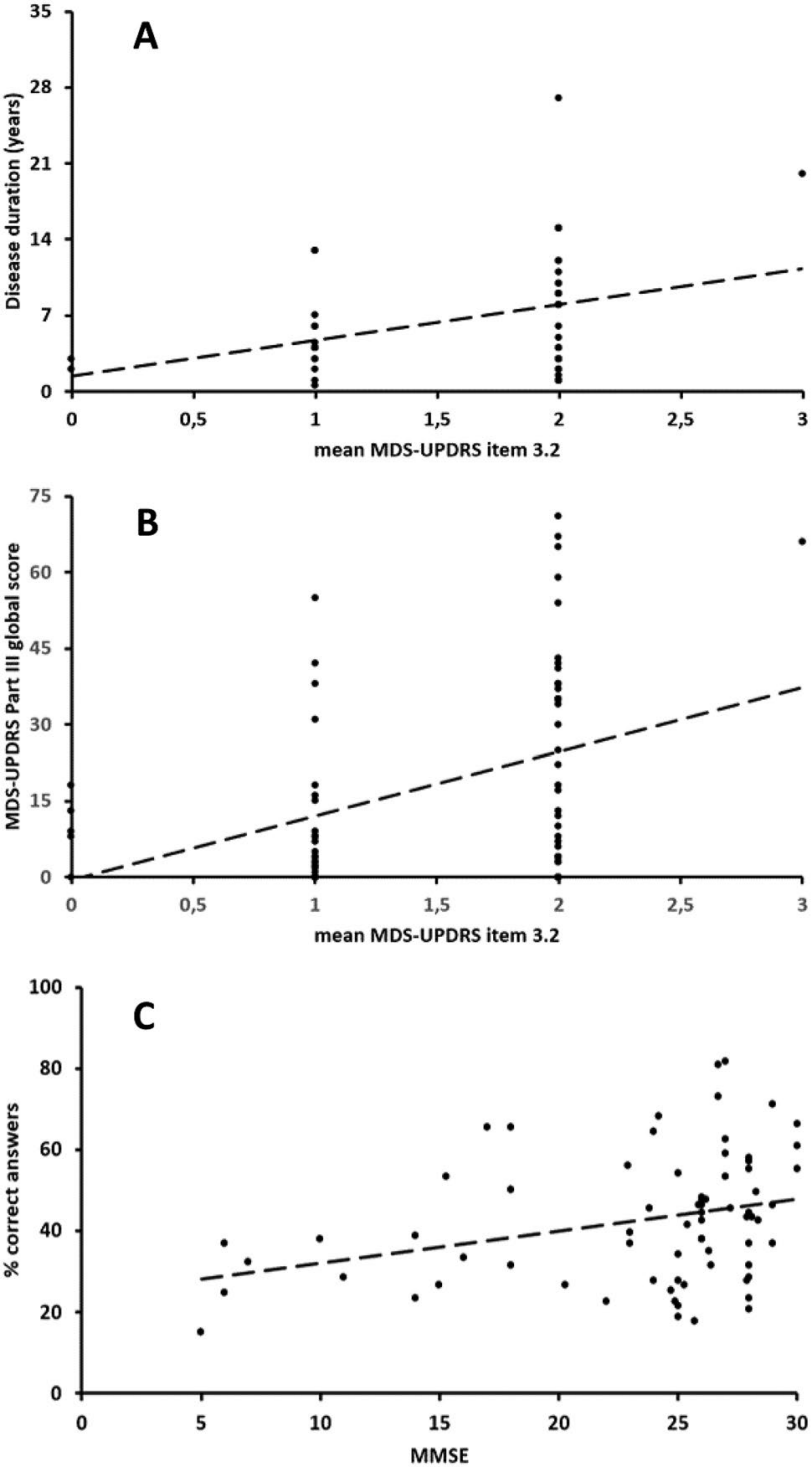
Correlation analysis. **A** Correlation between the Movement Disorders Society-Sponsored Revision of the Unified Parkinson’s Disease Rating Scale (MDS-UPDRS), Part III, item 3.2 scores and disease duration, expressed in years, in patients with PD and AD. **B** Correlation between the Movement Disorders Society-Sponsored Revision of the Unified Parkinson’s Disease Rating Scale (MDS-UPDRS), Part III, item 3.2 scores, and the global MDS-UPDRS part III scores in the three groups of participants. **C** Correlation analysis between the Mini-Mental State Examination (MMSE) scores and the mean percentage of correct answers given by the 15 raters on participants

A significant correlation emerged between the MDS-UPDRS-III item 3.2 scores and disease duration in PD and AD patients (*r* = 0.3, *p* = 0.03, Fig. 3A). Conversely, no correlation between the MDS-UPDRS-III item 3.2 scores and age emerged from the analysis. We found a significant correlation between the UPDRS-III item 3.2 scores and the total scores at the MDS-UPDRS part III in the three groups of participants (*r* = 0.35, *p* < 0.01), (Fig. 3B). Finally, a significant correlation emerged between the MMSE scores of the participants and the accuracy of the raters when rating the participants’ facial expressions (*r* = 0,34, *p* < 0,01), i.e., the subjects who had higher MMSE scores were those whose emotions were more easily recognized (Fig. 3C). No further significant correlations between demographic/clinical data and the overall recognition rate were identified in the analysis.

## Discussion

In the present study, we conducted a comprehensive assessment and comparison of facial expressivity in patients with PD, AD, and healthy individuals. The first original finding is that 58.4% (14/24) of patients with AD have reduced facial expression (hypomimia) in our sample. This result is in agreement with existing literature that suggests the presence of parkinsonian signs and symptoms, including hypomimia, bradykinesia, rigidity, postural instability, and gait abnormalities in AD (Soininen et al. 1992; Merello et al. 1994; Tsolaki et al. 2001; Tosto et al. 2016; Tangen et al. 2017; Roalf et al. 2018; Vöglein et al. 2019). Interestingly, we found a similar impairment in facial expressivity in PD and AD patients. Additionally, when analyzing individual emotions, no relevant differences were found in the pattern of expressivity of most of the emotions between the two patients’ groups, except for “surprise”, which was recognized in a lower percentage than in AD. The results obtained from this research have made it possible to develop some hypotheses about pathophysiological aspects of hypomimia in PD and AD, such as the involvement of specific neurotransmitter systems, brain areas, and higher neuronal functions, but also regarding diagnostic, clinical, and therapeutic implications.

Demographic characteristics, such as age, of the PD, AD and HCs groups were similar, so these factors did not influence our results. Gender distribution was similar between patients’ groups and HCs; however, we found a significantly higher prevalence of females in the AD group compared to the PD group, which should be acknowledged in the interpretation of our results. Cognitive performance was lower, as expected, in the AD group. However, based on clinical judgment, none of the patients had a cognitive status that did not allow them to perform the required task properly. AD patients also exhibited higher apathy scores compared to HCs, which could have potentially impacted the study outcomes. Nonetheless, we did not detect any significant differences in AES scores between AD and PD, nor between PD and HCs. Even though clinical criteria determined the diagnosis of PD and AD, all patients were consistently monitored in our outpatient clinic for several years, thus mitigating the possibility of a misdiagnosis. All participants were evaluated in the early morning. This methodological aspect minimized the effects of possible factors affecting the variability of the results, in particular blinking variability (Bologna et al. 2013; Ricciardi et al. 2017, 2020; Paparella et al. 2020, 2022). Again, to ensure that the evaluation of facial expressions was unbiased, raters were blind to the participants’ diagnoses, and both the videos and the pictures were presented in random order. Finally, to avoid any external influence on the assessments, the video frames focused only on the subject’s head, thus eliminating the possibility that additional neurological symptoms, such as tremors or altered body posture, would influence the judgment.

The observation that both PD and AD display a similar pattern of reduced facial expression, without emotion-specific differences in facial expressions, supports the hypothesis of a shared mechanism underlying this phenomenon in both pathological conditions. One possibility is that the diminished facial expression in PD and AD is attributable to a central dopaminergic deficit. Experimental evidence suggests a potential link between the reduction in central dopaminergic activity and hypomimia in PD. For instance, it has been noted that patients with hypomimia tend to exhibit higher MDS-UPDRS-III scores during the OFF state, primarily due to bradykinesia and rigidity (Ricciardi et al. 2020; Sampedro et al. 2022) and that hypomimia is a levodopa-responsive feature in PD alongside other motor symptoms (Ricciardi et al 2020). Furthermore, patients experiencing hypomimia commonly demonstrate a higher level of apathy, which is another symptom related to dopaminergic dysfunction, compared to individuals without hypomimia (McGuigan et al. 2019). However, in the current study, we did not identify any statistically significant differences in the severity of apathy when comparing individuals with and without hypomimia. Therefore, this aspect requires further clarification. Regarding AD, evidence indicates a potential deficiency in central dopaminergic circuits. In this regard, a recent meta-analysis has yielded significant evidence of a substantial reduction in dopamine, D1R, and D2R concentration levels among AD patients compared to controls (Pan et al. 2019). Moreover, the reduction in dopamine levels and dopaminergic receptor expression has been found to correlate with the severity of AD to some extent. Experimental studies have further revealed that neurons within the nigrostriatal pathway can undergo various pathological changes, including the accumulation of β-Amyloid plaques, leading to neuronal loss and decreased central dopaminergic tone (Pan et al. 2019). These findings collectively support the idea of a dopaminergic deficit as a common pathophysiological mechanism in PD and AD for hypomimia. However, differently from PD, it should be acknowledged that the hypothesis linking AD-related pathological processes, dopaminergic dysfunction, and the observed decline in facial expressiveness in this condition remains speculative (Perez et al. 2005), and further prospective studies are needed to clarify this relationship. When considering the hypothesis of dopaminergic deficiency as the cause of reduced facial expressiveness in both PD and AD, it is important to acknowledge that some of our observations might not completely align with this hypothesis. For example, the AD patients in our study did show only subtle parkinsonian signs, and their MDS-UPDRS scores were lower compared to the PD patient group. This would imply that dopaminergic deficiency in AD would affect early and predominantly facial district movements. This hypothesis could be explained by the lack of “visual feedback” in hypomimia that could recognize, control and intervene through compensatory mechanisms in modifying facial bradykinesia or through those mechanisms that have been shown to be effective in improving limb bradykinesia (Bonassi et al. 2016). The absence of compensatory mechanisms capable of “hiding” the presence of alterations in facial expressiveness might suggest hypomimia as an excellent biomarker to track and diagnose early neurodegenerative processes. Aside from the dopaminergic hypothesis, additional neurotransmission systems may play a role in the development of hypomimia in our patients, notably the cholinergic system. In this regard, recent evidence has indicated the role of altered cholinergic neurotransmission in motor impairment among individuals with AD (Schirinzi et al. 2018; Bologna et al. 2020), as well as in cognitive disturbance in PD (Zenuni et al. 2023).

Again, when exploring the potential underlying pathophysiological basis of hypomimia in both PD and AD, the involvement of specific brain structures in the limbic areas, particularly the amygdala, which are believed to play a significant role in emotion processing (Harding et al. 2002; Argaud et al. 2018b; Ferrari et al. 2021) needs to be considered. Indeed, it is widely recognized that although AD and PD have distinct anatomo-pathological substrates, both conditions can affect the limbic structures. In this context, the presence of cortical atrophy in these regions may be linked to the severity of the impairment in the ability to perceive facial expressions and hypomimia (Baggio et al. 2012; Junqué et al. 2005; Ibarretxe-Bilbao et al. 2009; Ramírez-Ruiz et al. 2005).

Additionally, in support of the idea that hypomimia in PD and AD does not necessarily reflect a mere motor impairment, there is evidence suggesting that more pronounced hypomimia can be observed in both PD and Mild Cognitive Impairment of the AD type, regardless of the severity of motor impairment or motor phenotype. This observation indicates that the altered facial expression may be partly linked to the impairment of higher brain functions (Gasca-Salas and Urso 2020). This hypothesis also highlights the possibility that the reduced facial expression in AD and PD may arise from various mechanisms, with differential contributions of specific pathways in each condition. In this regard, it should be acknowledged that reduced facial expressivity in AD may reflect, at least in part, altered face praxis functions. Facial apraxia has been recognized in different dementia syndromes, including AD and frontotemporal dementia (Capone et al. 2003; Yliranta and Jehkonen 2020), and different apraxia profiles can also allow distinguishing among the various subtypes of dementia (Johnen et al. 2016; Yliranta and Jehkonen 2020). Moreover, recent evidence demonstrated that specific clinical apraxia tests, including face praxis evaluation, can help detect AD from the earliest disease stage (Yliranta et al. 2023).

The hypothesis that hypomimia represents an intrinsic manifestation of both diseases has pathophysiological and clinical implications. Especially, the results in this study showed a significant correlation between the severity of hypomimia and disease duration, but not with the age of the subjects. This could indicate that the reduction in facial expressiveness represents an intrinsic condition of both diseases, which can be objectified from the earliest stages of the disease (Sampedro et al. 2022) and tends to be more pronounced proportionally to the neurodegenerative process, regardless of the subject’s age. Furthermore, a greater degree of hypomimia correlates significantly with greater motor impairment as assessed by MDS-UPDRS part III, which could indicate how these symptoms may represent the expression of intrinsically related causal mechanisms (Sampedro et al. 2022). Moreover, the correlation between greater accuracy of raters in the assessment of facial expressions and higher scores on the MMSE would seem to support the hypothesis of an involvement of higher brain functions in which cognitive aspects, likely cortical, play a role in hypomimia.

Hypomimia in patients with AD or PD has significant clinical implications. In fact, the results of this study suggest that the presence of hypomimia, a neurological sign traditionally associated with PD, even if in association with other parkinsonian signs, does not, however, allow a diagnosis of AD to be completely excluded. Moreover, the assessment of facial expressiveness during the progression of AD and PD is crucial, as hypomimia emerges early in both conditions. This opens the space for new prospectives in which technological tools that enable mass screening for early detection of neurodegenerative diseases may help in this regard. In addition, it is essential to explore the potential relationship between hypomimia and its impact on daily activities. In people with AD, alterations in facial expressiveness can lead to negative perceptions and judgments by caregivers and health care providers, negatively impacting the quality of interpersonal interactions and medical care.

The present study has some limitations that should be acknowledged. Firstly, a possible limitation could be the different gender distribution in the three groups, with a higher prevalence of female subjects within the AD group. This discordance is due to the consecutive recruitment of study participants. However, no significant difference emerged when both groups of AD and PD patients were compared with HCs and, in addition, there are no previous literature data that have highlighted the presence of gender differences in hypomimia, therefore, it is improbable that this factor represents a real limitation on the validity of the results of this study. The disease duration was also different between PD and AD, with the former group exhibiting a longer duration. While it is essential to consider this finding in conjunction with the positive correlation we identified between hypomimia and disease duration, it is also important to acknowledge that a direct comparison between these two neurodegenerative diseases is not feasible due to their distinct rate of progression. Moreover, the relatively small sample size may have impacted the generalizability of the results. Additionally, PD and AD diagnoses were based on clinical criteria, and the lack of a biological diagnosis, especially for AD, might represent a study limitation. Furthermore, none of the patients with AD undergo DAT scans to assess dopaminergic function. While the assessments were conducted in the morning before patients took their regular medications, we cannot entirely rule out the potential influence of drugs that act on the central nervous system, which could serve as possible confounding factors in our patient group. Additionally, it is worth noting the possibility of a long-lasting effect of levodopa in the PD group. In addition, the inter-raters’ agreement was slight. This result could be explained by the fact that there is not a standardized method for assessing the features and degree of hypomimia, taking into consideration that any proposed clinical scale unfortunately has a certain level of approximation. Finally, the assessment of facial expressivity of emotions in individuals with PD was limited to the OFF state; therefore, the influence of dopaminergic therapy on facial expressiveness has not been evaluated.

In conclusion, this study has provided novel evidence that the impairment of facial expression of emotions is a shared and nonspecific neurological sign in the prevalent chronic neurodegenerative diseases, namely AD and PD. The deficit in emotional expressiveness observed in these conditions can hold significant pathophysiological implications, although further experimental research is required to delve deeper into this area. Namely, further studies are required to explore the potential connections between reduced facial expressiveness and aspects related to the processing of emotional stimuli, as well as the broader domain of mood and higher cognitive functions. These future investigations would enhance our understanding of the underlying mechanisms and offer valuable insights into the emotional and cognitive aspects associated with diminished facial expressiveness in AD and PD. Additionally, it may have noteworthy clinical and diagnostic implications, and impact the overall quality of life.

## Data Availability

The research data associated with a paper is available on reasonable requests.

## Abbreviations

AD: Alzheimer’s disease
ADL: Activities of Daily Living
AES: Apathy Evaluation Scale
BAI: Beck Anxiety Inventory
BDI: Beck Depression Inventory
CL: Confidence Level
FAB: Frontal Assessment Battery
FSS: Fatigue Severity Scale
HCs: Healthy controls
IADL: Instrumental Activities of Daily Living
MMSE: Mini-Mental State Examination
MDS-UPDRS-III: Movement Disorders Society-Sponsored Revision of the Unified Parkinson’s Disease Rating Scale - Part III
PD: Parkinson’s disease
RT: Reaction time
SD: Standard deviation
